# Wastage in the health workforce: some perspectives from African countries

**DOI:** 10.1186/1478-4491-3-6

**Published:** 2005-08-10

**Authors:** Delanyo Dovlo

**Affiliations:** 1 Population Council, Accra, Ghana

## Abstract

**Background:**

Sub-Saharan Africa faces a human resources crisis in the health sector. Over the past two decades its population has increased substantially, with a significant rise in the disease burden due to HIV/AIDS and recurrent communicable diseases and an increased incidence of noncommunicable diseases. This increased demand for health services is met with a rather low supply of health workers, but this notwithstanding, sub-Saharan African countries also experience significant wastage of their human resources stock.

**Methods:**

This paper is a desk review to illustrate suggestions that the way human resources for health (HRH) are trained and deployed in Africa does not enhance productivity and that countries are unable to realize the full potential expected from the working life of their health workers. The paper suggests data types for use in measuring various forms of "wastage".

**Results:**

"Direct" wastage – or avoidable increases in loss of staff through factors such as emigration and death – is on the rise, perhaps as a result of the HIV/AIDS epidemic. "Indirect" wastage – which is the result of losses in output and productivity from health professionals' misapplied skills, absenteeism, poor support and lack of supervision – is also common.

HIV/AIDS represents a special cause of wastage in Africa. Deaths of health workers, fear of infection, burnout, absenteeism, heavy workloads and stress affect productivity.

**Conclusion:**

The paper reviews strategies that have been proposed and/or implemented. It suggests areas needing further attention, including: developing and using indicators for monitoring and managing wastage; enhancing motivation and morale of health workers; protecting and valuing the health worker with enhanced occupational safety and welfare systems; and establishing the moral leadership to effectively tackle HIV/AIDS and the brain drain.

## Introduction

Africa, unlike the other continents, faces a severe human resources crisis in the health sector. The continent's economic performance has been poor, which has affected the ability of countries in sub-Saharan Africa (with few exceptions) to sustain credible health services and to train, employ and use health workers most efficiently. Economic growth has been low or negative in many countries, with investment in health that has generally been inadequate, both as proportions of GDP and in gross terms. Motivation, incentives and productivity and retention of health workers have been severely affected. Furthermore, over the past two decades the population of countries in the SSA region has increased significantly, with a major expansion in the disease burden due to HIV/AIDS, recurring high levels of communicable diseases and recent rises in the incidence of noncommunicable diseases and other diseases related to diet and lifestyle changes. However, in the face of the high demand for health services that the foregoing entailed, sub-Saharan Africa has had a low supply of health workers, and this notwithstanding, also experiences significant wastage of its human resources.

While recognizing the paucity of health workers in Africa and the retention and motivation difficulties, this paper suggests that the way human resources in health are trained, deployed and managed by many countries in the region reduces their productivity. Thus these countries are unable to realize the full potential that could be reasonably expected from the working life of their health workers. The potential of health workers to produce health, even within the constraints alluded to, is often shortened by severe attrition and other more indirect forms of "wastage".

For the purposes of this paper, the author uses the term "wastage" to refer to the loss in utility of health workers/health professionals due to attrition or poor productivity that can be prevented or managed and that is over and above what is expected in normal work situations.

Wastage of human resources may be seen from a variety of viewpoints. In some countries, wastage may result from underuse or non-use of trained personnel, resulting in unemployment caused by overproduction, retrenchment or an inability to absorb certain skill types. Others experience wastage as when a health system is unable to realize the full potential and skills of its health workers even when they are fully employed; this second area of wastage is felt to be a problem for a number of health systems in sub-Saharan Africa. Wastage thus goes beyond mere attrition or the losses, normal or otherwise, that occur within a workforce.

For the purposes of this review and based on the foregoing definition, "wastage" has been classified into two main forms: "direct" and "indirect".

"Direct" wastage occurs when avoidable loss of health personnel arises from factors such as emigration and death (i.e. complete losses to the health sector) and reflects attrition of people from the health workforce. "Indirect" wastage is the result of losses in output and productivity of health professionals, such as those arising from absenteeism and poor performance.

HIV/AIDS is discussed in this paper as a special cause of wastage with combined effects of both direct and indirect wastage. Though various aspects of the impact of HIV/AIDS on health workers reflect either direct or indirect losses, the severity of its effects merits separate attention. Deaths of health workers have risen exponentially in some countries in recent years, and many health workers may be leaving the workforce from fear of infection. Burnout, absenteeism and stress among staff are other effects of HIV/AIDS.

The premise of our discussion is therefore that the health workforce in health in Africa faces many challenges and is in a crisis; countries face the following:

• Preventable exit of professionals from the workforce is a major wastage. Deaths, early retirement, emigration and retrenchment have also shortened the optimal working life of health workers.

• Excess production of some types of health workers has occurred without adequate use or with unemployment or underemployment.

• The supply of health workers may at times not match required skills and scopes of practice, which in turn may not match service delivery needs.

• Poor human resources management results in suboptimal deployment and use of professionals.

• Staff time may be inappropriately applied: for example, in some countries a heavy load of in-service training activities and general administrative duties for technical staff reduces the time available for service delivery.

The method used was to review publications, conference reports, presentations at meetings and other data/information on human resources in health in Africa. The wastage issues raised that were related to the five types of losses mentioned above were then categorized and discussed. Because of the paucity of data from countries on the continent, peer-reviewed papers as well as unpublished country reports and communications were reviewed and analysed.

This paper is intended to help identify and clarify causes of wastage and discuss possible indicators that may assist health care managers to monitor, manage and reduce the various forms of wastage. Using experiences from sub-Saharan Africa, wastage of health workers is illustrated by factors such as increased pre-retirement mortality, early and premature retirement, increased emigration and high levels of workplace accidents and injury. While the review did not determine any current standards or benchmarks that exist to show routinely expected levels for such wastage, trends and comparisons between similar countries point out some problem areas. In the following sections, the concept and evidence of different forms of wastage are discussed, using experiences and information from African countries.

## Discussion

In this section, the paper examines the various modes of the two types of wastage defined above and illustrates some of the situations in which these occur. In addition, the paper further reviews, as an exceptional case, the impact of HIV/AIDS on wastage of human resources for health.

### Direct wastage

Attrition of workers from any form of employment is an expected factor in human resource management, as workers change jobs, retire or die. However, if for any reason the rate of attrition is higher than normally anticipated, this may reflect a problem.

"Direct wastage" as discussed here represents those losses from the stock of health professionals that are considered to be over and above the norm. For example, the total workforce or a component of it (such as nurses) may be expected to lose members at about 1% of stock per annum. If this rate of losses rises suddenly or changes significantly over time, a retention problem may be indicated. In the case of deaths, a health problem may be indicated.

These changes (increased attrition) may occur over time within the same country or as differences between the attrition rates of two similar countries and their populations of professionals. Direct wastages can present in many forms, and from this review some of the wastage types encountered are represented below.

### Movement from health into non-health professions

Health professionals may leave health work altogether and do something completely unrelated. While those who do this have not been fully studied in the literature reviewed, they appear to be a small proportion of the loss of professionals. Dovlo and Nyonator (1999), studying a cohort of 192 doctors in Ghana, found that only two had changed professions completely (had became full-time ministers of religion) [1]. The Mozambique Health Ministry (2003) said 18 nurses changed jobs in 2002, of whom five retrained as doctors and others went into psychology, law, biology, international affairs and geography [2]. A 1999 study of health worker migration showed that the proportion of nurses leaving the workforce who chose to leave before their working life ended ranged from 23% to 78% of all leavers depending on the country [3]. Table 1 shows the proportion of staff departing from the health workforce who leave public sector employment prematurely in some African countries. There is no indication as to whether these leavers remain in health work or within the country. Work done for the Joint Learning Initiative on Human Resources for Health by Dare et al. [4] (also indicated that 7.9% of doctors in Nigeria worked outside the health sector.

**Table 1:** Voluntary leavers: example of direct wastage in selected countries, 1999

**Categories**	**Ghana**		**Lesotho**		**Namibia**		**Malawi**	
	Total leavers	% voluntary	Total leavers	% voluntary	Total leavers	% voluntary	Total leavers	% voluntary
Nurses	744	58.7%	NA	NA	47	78%	43	23%
Doctors	315	84.4%	NA	NA	16	93.8%	18	83.3%
Others/All	-	-	50	62%	-	-	17	76.5%

Source: Dovlo D: *Issues Affecting the Mobility and Retention of Health Workers/Professionals in Commonwealth African States*. A consultancy report prepared for the Commonwealth Secretariat. London; 1999.

### Emigration/brain drain of health professionals

Significant increases in the migration of health professionals have occurred in recent years but monitoring of emigration flows is difficult, as few countries keep adequate statistics. Dovlo and Nyonator (1999) estimated that between 1986 and 1995, 61% of doctors who qualified from one medical school in Ghana left the country [1]. Of these, 6.2% had migrated to another African country (South Africa), but most went to the United Kingdom (55%) or the United States of America (35%). Huddart and Picazo (2003) indicate that 840 out of 1200 doctors trained in Zimbabwe in the 1990s left the country and 17% of locally trained physicians and dentists left the Sudan in the 1980s and 1990s [5]. In the case of Ghana, the physicians had left within 10 years of qualification, after working less than a third of the expected duration of their services.

While the numbers emigrating are in themselves problematic, they also hide serious qualitative consequences that occur when losses of even small numbers of specialists and tutors create a much wider effect on the training of new health workers and in sustaining quality. For example, Martineau and Decker (2002) report that the recruitment of just two specialized anaesthetists from the Boxburg Centre for Spinal Injuries in South Africa by a Canadian Institution led to permanent closure of the unit [6].

In smaller countries, even minimal numbers of migrants represent significant losses. In relatively wealthy Mauritius, the Ministry of Health estimated that 327 nurses (about 12.9% of the nurse workforce) migrated from an average annual nurse workforce level of 2534 between 1998 and 2001. 89% went to the United Kingdom, 8% to New Zealand and 3% to Canada and the United Arab Emirates [7]. While the impact of these losses has not been thoroughly studied, it has been suggested that migration means a loss of the investment made in health workforce development, which creates equity and distribution problems within the country's health systems and causes high workloads, poor quality of care and low morale among health workers [8,9].

### Work-induced injury, accidents, deaths as causes of premature loss from the workforce

The past decade has shown marked increases in death rates of health workers in some African countries. Huddart and Picazo (2003) and Tawfik and Kinoti (2003), among others, have ascribed these deaths to the silent impact of HIV and AIDS on the health workforce [5,10]. However, African health systems can also be blamed for work environments that induce high levels of occupational accidents or create a strong perception of high risk [11]. The Chief Nursing Officer of the Ghana Health Service has suggested at a national health workforce forum that high workload and stress may have contributed to higher than usual trends in nurses' deaths (an approximate 26% rise in nurse deaths between 2001 and 2003) in a country with comparatively low HIV prevalence rates.

A study of nurses and teachers in Ghana (Clarke, 2003) showed cervical spondylosis as the second most common cause of morbidity among nurses, with nurses being 21.5 times more likely to develop lower back pain than teachers and 1.4 times more high back pain [11]. Despite the lack of benchmarks on the expected levels of morbidity and mortality, the rising trends in death and disability over the years depicts a serious problem.

HIV/AIDS will be discussed later in this review, but it is mentioned here as a major contributor to "direct wastage" due to high death rates being reported among health workers. A 1999 study found that among workers leaving public health services in Malawi, 25% of clinical officers and 51% of nurses leaving had died, compared to deaths constituting 1.1% deaths among all staff leaving Ghana's Ministry of Health. Recent Government of Malawi/United Nations Development Programme work has been quoted in Aitken and Kemp (2003) that shows deaths as the main cause of losses from the Malawi health workforce [12].

### Inefficient personnel administration

Health workforce management in many African countries is part of civil service administration. For example, it was found that in Lesotho, recruitment delays meant new health workers at times spent a year before being appointed and sometimes an entire batch of new nurses is lost to the public service due to delays in processing their appointment [3]. Moreover, structural adjustment policies aimed at reducing public sector expenditures have led to retrenchments and a recruitment freeze in countries such as Cameroon and Uganda, even in the face of low availability of health workers and poor coverage of health services [13].

Ghana and Zambia have tried to address management deficiencies by "de-linking" health services from the Civil Service and creating new autonomous agencies (Ghana Health Service, Zambia Central Board of Health). In Ghana, the de-linked agencies (two tertiary hospitals and a Ghana Health Service) have been established and are gradually extending their autonomy. In Zambia anecdotal evidence suggests this experiment has not worked out well for several reasons related to transfer of workers' benefits and pensions to the new agency. The literature is not available to indicate whether implementing these structures has improved health workforce management or not, and given the available migration data, they have not improved retention and motivation of staff.

### Indirect wastage

The concept of indirect wastage as contrasted to direct wastage implies losses that arise from inefficient productivity or use of health workers. Such waste of human resources is the result of the inefficient or poor use of staff already employed and providing services. Other forms of indirect waste include the inappropriate use of skills, and "ghosts" that plague payrolls while restricting room for new employment.

Almost all the issues discussed in this section border on effective management systems for human resources, but the contentions raised below elaborate on some aspects of indirect wastage of staff derived from examples in African countries. For example, absenteeism rates in similar groups of health workers (within the same country or in different countries) may vary significantly, indicating a wastage problem in one group. On the other hand, using available professionals for work not related to their skills represents another form of wastage, especially if shortages of those skills exist in the country.

### Wastage as unemployment of available staff

In sub-Saharan Africa, even with its well-acknowledged shortages of health workers, unemployment occurs. Ngufor (1999) in Cameroon suggested that structural adjustment policies and related fiscal limits on governments have meant that new health graduates are not employed even when the demand exists; instead, retrenchments from the public sector continued to be encouraged [13]. Personal communications from a Deputy Commissioner of Health for Human Resources Development in Uganda also confirmed this problem.

A second factor is the blockage of health worker positions by "ghost workers" – persons who fill the payrolls but do not actually exist at workplaces. A recent report on case studies of African countries commissioned by the High Level Forum on the Millennium Development Goals found, for example, that Kenya had some 5000 ghost workers on its payroll [14].

### Wastage as ineffective staff use

Underuse often occurs when staffing norms and established posts in the public service do not relate to actual workload needs but are standardized by facility type. Underemployment and underuse may result in facilities with widely varying patient loads having the same staff strength, with redundant staff in some areas and overworked ones in others.

Along with underuse is some level of misuse. Delegates from Malawi attending a migration conference in South Africa suggested that trained midwives may be avoiding postings into labour and delivery wards for fear of possible risk of exposure to HIV-infected blood. Thus less-qualified staff members are left to offer the services instead [15]. The Ghana Health Service, for example, employs 5.3% of all its doctors in mainly managerial functions at its administrative headquarters, while the deprived Upper-West Region, with a population of about 600 000, has only 1.5%, or 10 doctors [16]. Thus the use of health professionals in administrative roles or in inappropriate duties represents a form of wastage of the available resources.

### Wastage resulting from poor skills/cadre mix

Ghana, Kenya, Lesotho, Malawi and Zambia have banned enrolled nurse training, even when significantly increased migration of registered nurses has severely reduced the nursing workforce. Enrolled nurses usually received two years' or less formal training and entered nursing training with lower qualifications, while registered nurses normally completed 12 years' basic education and received 3 or 4 years' professional training. Enrolled nurses usually served as auxiliaries to registered nurses. The result is a workforce that is highly internationally mobile and costs more to remunerate, while being made to carry out some tasks that enrolled nurses could readily do. The use of other mid-level health workers has often been limited by restrictions in scope of practice and resistance from the more established professional groups.

Again, despite the high need for rural health services and the high migration rate among its doctors, the health sector in Ghana produces six times more doctors annually than medical assistants, who are better retained and are more likely to be found in rural areas [17].

### Wastage arising from low health worker performance and outputs

The volume and quality of work expected from otherwise competent staff are not always forthcoming, and many reports exist of absenteeism and low productivity among health workers. The main referral hospital in Ghana (Korle-Bu Teaching Hospital) in its 2002 annual report, recorded 1334 days of sick leave in 2002 by 556 nurses from a total nurse workforce of 809. Thus 70% of the nursing workforce reported ill at some time during the year, an average of 2.4 days off per nurse [18]. Staff time is also lost writing reports and carrying out basic administrative tasks. Absenteeism from HIV/AIDS is discussed separately.

The large amount of in-service training carried out by various programmes is recognized as one of the sources of wastage through sanctioned absences of staff. Though training provides skills and may arguably enhance productivity, it has sometimes provided an inverse incentive in poor countries due to generous allowances received as participants. Key staff members spend a lot of time in training courses organized by various agencies and programmes.

We had earlier under direct wastage recognized the role that the migration of trainers and specialists plays in mitigating the effectiveness of the remaining workforce's productivity. Achieving viable productivity requires a good mix of professions, with adequate numbers providing good supervision.

### Wastage from misadministration of human resources

The poor management of human resources for health found in many sub-Saharan African countries is likely to contribute to widespread misdeployment and maldistribution. It contributes directly to wastage in specific ways. "Ghost" workers are a problem in many African countries, as health workforce administrators lack good staff databases and payroll systems are poorly managed. Poorly regulated or unregulated dual practice carried out by public sector doctors and nurses working in the private sector may lead to neglect of their government duties. Poor support and supervision of health workers was cited as a problem in many African countries at the Commonwealth Workshop on Developing Strategies for Attracting and Retaining Health Workers in January 2003.

Distribution problems in Ghana, for example, have resulted in the country's three most deprived regions – all with serious maternal mortality problems – having only a single gynaecologist and two surgeons serving one third of the country's land area and one sixth of its population, while 35% of all health staff are found in just two teaching hospitals [19]. Distribution problems are common, with rural and periurban slum communities probably the most deprived of trained professionals.

### HIV/AIDS and HRH – a special case of wastage

The impact of HIV/AIDS on the workforce, as alluded to previously, creates both direct and indirect forms of wastage. Its complex and self-reinforcing negative impact on the health workforce merits specific mention as a major emerging source of HRH wastage.

A study by Buve et al. in Zambia showed that mortality rate among female nurses in two hospitals rose from between 2 per 1000 in 1980–1985 to 26.7 per 1000 in 1989–1991 [20]. World Bank projections quoted by Kinoti (2003) are that a country with 15% adult seroprevalence rate for HIV can expect to lose between 1.6 and 3.3% of its health care providers from AIDS annually, a direct wastage [10].

However, indirect wastage from HIV/AIDS can have as bad an effect as the direct wastage noted above. Kinoti and Tawfik (2003) estimate that absenteeism can consume up to 50% of staff time in the final year of life for health workers with AIDS. Calculations from Botswana showed that if the average infected health worker lost 60 working days in his or her final year of life, this would translate into a loss by the public health sector of 23 000 workdays in 2003 alone [21]. This excludes absenteeism arising from workers needing to attend numerous funerals of relatives and co-workers and other forms of indirect wastage.

Few countries in sub-Saharan Africa appear to have instituted programmes to cater for the counseling, support and antiretroviral treatment needs of health workers. A recent press release reporting collaboration between International Council of Nurses, Zambian Nursing Association and the pharmaceutical firm Boehringer Ingelheim to supply nevirapine to health workers is one of the new initiatives that need to be expanded quickly [22].

Managing the impact of HIV/AIDS on the health workforce in high-prevalence countries must necessarily be an important aspect of reducing both direct and indirect wastage and improving productivity from health professionals. The huge need that will be generated by the global initiative to treat 3 million persons with antiretroviral drugs by 2005 may further encumber the existing workforce away from routine duties that are not tied to a specific project.

## Reducing wastage and improving staff retention

Table 2 proposes indicators for health worker wastage as a framework that country health workforce managers may use in monitoring the extent of various forms of wastage. Indicators for direct wastage depend on having a fairly robust human resources information system or the ability to carry out surveys from time to time to determine trends.

**Table 2:** Wastage monitoring framework

**DIRECT WASTAGE**		
**Factor**	**Examples of contribution to wastage**	**Possible indicators**

Movement from health to non-health sector	Probably small: 2 – 20 staff per year (Ghana. Mozambique, Namibia)	% of job leavers exiting health work completely (exit interviews)
Emigration to health sector outside country	10% of Mauritian nurses, 61% of Ghanaian doctors	Certificate verification rates Routine leaving data, e.g. resignations
Deaths, injuries and premature removal from the workforce	High significance of HIV/AIDS; Ghana 1.1% deaths compared with Malawi (<55%) of leavers	Mortality rates as % of workforce leavers, or Mortality rate in workforce
Inappropriate Administrative systems and policies	Affects other losses. Delays lose work input and may increase likelihood of emigration.	Average recruitment duration Staff recruitment rate versus vacancies

**INDIRECT WASTAGE**		

Wastage as unemployment	Not well documented in Africa. Estimates of "ghost workers"?	Unemployed health workers as % of total workforce (for each category)
Wastage as underemployment	Data is not routinely collated but staff/workload indicators may help.	Staff workload Indicators, e.g. outpatient and inpatient staff per cadre
Wastage as a misuse	Significant in countries with senior medics and nurses as managers.	% staff: technical or professional in full-time managerial/administrative function
Wastage as inappropriate categories	4–6 categories to deliver package of services in Ghana.	Workforce composition of skilled and semi-skilled staff
Absenteeism, low outputs	2.3 days' sick leave per staff member versus 1.65 days off for all staff (Ghana)	Number of days off per staff member, per annum.
Misdeployment and maldistribution	Distribution differential: Doctors (Ghana): best 1:16201, worst 1:66071	Doctor/nurse population ratios in different parts of country.
Wastage from misadministration of HRH	Difficult to assess quantitatively: e.g. 100% of new Lesotho nurses not recruited in 1998	Recruitment and retention rates of new graduates of health training schools.

In our study, numerator difficulties have sometimes made the use of data for indicators difficult. While we could determine losses from the workforce through civil service statistics, the numbers of workers actually in the workforce, for example, was more difficult to determine with accuracy. However, the number of deaths as a proportion of the total number of people leaving public service is clearly rising; this paper suggests this could be a fairly simple system of monitoring such changes. In some countries, the reasons for leaving the workforce are not recorded in much detail; using exit interviews or forms is recommended as a way to collect data.

"Ghost" workers are a problem in some SSA countries, where the names of nonexistent workers are maintained on health payrolls by dishonest managers. It was difficult to think of routine ways to monitor "ghost" workers, apart from conducting snap censuses at workplaces, by means of the payroll. These snap censuses will probably not work as tools for regular routine implementation.

Indicators for indirect forms of wastage are much less categorical and more complex than those for direct wastage. For example, given the difficulties with data on employed health workers it might be difficult to determine how many are unemployed. This information could be collected from census data, but censuses take place at long intervals. Skill mix also represents a challenge, as the standards vary widely between countries and not many standardized benchmarks exist. Again, this may best be served by surveys showing trends and changes rather than measurements against a particular standard.

Workload standards are important to determine underemployment and appropriate distribution and deployment of health workers. Indicators can then be prepared to match staffing levels with workload. A difficulty here is that workload may vary according to seasons or with other factors, and thus workload data must be observed and analysed for a period before major changes are made. For example, attendance at health facilities may be very high during harvest periods when migrant labour comes to assist with harvest and accidents occur frequently. It may also vary over time with changes in population and economic activity in the area.

Using data to manage wastage is useful only if management systems are coherent and countries attempt to put in place strategies to cope with wastage in a comprehensive way. A number of coping strategies have been tried and many more proposed. Strategies implemented have included improving incentives and motivation of health workers through various mechanisms. In Ghana, incentives include new extra duty allowances, vehicle loans, cash incentives for rural based health workers, and local specialist training opportunities. On the other hand, Eritrea exacts a 2% income tax on its citizens living abroad; such remittances are at the level of 85.8% of development aid received. Other countries, such as Nigeria, receive remittances from their émigré community as significant sources of foreign exchange, far outstripping official development aid [23].

Using clinical officers and medical assistants to deputize for doctors may be disputed, but these categories are less internationally mobile and do mitigate shortages caused by emigrating doctors, provide comparable quality of service and are more likely to serve and remain in rural areas [24]. Some countries have recruited doctors from Cuba and some others recruit significant numbers of health workers from other African countries.

This paper has not dealt with the movements between the public and private health sectors within countries, as these are deemed to be part of the health system and are not included in wastage. Concerns do exist, however, about the possible neglect of rural areas in siting of private health facilities and hence the pull of health workers to urban areas away from other geographical zones with more needs. Moreover, dual practice by public sector health workers in the private sector may well induce wastage, as neglect of their public sector duties may ensue as a result. Private sector health professionals were most significant in Kenya, Nigeria and South Africa, among other countries.

## Conclusion

Caught in a vicious cycle, the poor African economies are unable to fund systems to manage and control wastage adequately, even as new international investment in the health sector has increased demands on staff while restricting investment in incentives. The HIV/AIDS epidemic, combined with the economic crises, threatens even the few coping mechanisms that are being attempted [25].

### What can be done to alleviate the problem?

The capacity of countries' HRH departments must be strengthened, and development partners and governments must invest significant portions of health budgets in building capacity, not only through training, tools and technology, but with incentives to retain staff. Currently, most health sector human resources departments are managed as part of the general civil service and have little influence on policy development; they may lack specialists in health workforce planning and management.

Awareness of the problems of wastage can be increased by integrating wastage indicators into human resources information systems used by country HRH managers. Some indicators have been suggested in Table 2; structuring data systems to collect and analyse these indicators may provide evidence to compare productivity of different health service delivery units and also to advocate changes in how human resources for health are planned for and managed.

### Motivation and morale are key factors in wastage. How can this change?

Governance and leadership in health must now be expressed as tangible actions that result in senior managers and policy-makers valuing and respecting health workers. New career and incentives systems must be developed, along with better social and technical support for health workers. Real or perceived occupational risk from the health workplace appears to contribute significantly to low morale and consequent wastage. The public sector must establish occupational health services that assure prevention and treatment for workplace incidents. An essential component of this service should include voluntary counseling and testing, as health workers also need education on HIV/AIDS as well as the antiretroviral treatment policies that are becoming more available in other industries.

### Is moral leadership needed?

There is almost a sense of helplessness in dealing with the HRH crisis in Africa. Because health managers anticipate that development partners will avoid support for HRH incentive issues, they now rarely include them in their proposals to the global funding agencies. Emigration is another area where international action has mainly been in the form of voluntary codes of conduct that have had little effect. Economic, labour market and human rights arguments are made by the developed countries as the basis for their reluctance to assist developing countries to manage emigration more effectively. However, the HRH crisis in Africa requires that countries on both sides also create a moral discourse to take actions that will improve the health of Africans.

## Competing interests

The author(s) declare that they have no competing interests.

## Authors' contributions

Delanyo Dovlo was the sole author.
